# Postnatal gestational age estimation of newborns using Small Sample Deep Learning^☆^

**DOI:** 10.1016/j.imavis.2018.09.003

**Published:** 2019

**Authors:** Mercedes Torres Torres, Michel Valstar, Caroline Henry, Carole Ward, Don Sharkey

**Affiliations:** a School of Computer Science, University of Nottingham, United Kingdom of Great Britain and Northern Ireland; b School of Medicine, University of Nottingham, United Kingdom of Great Britain and Northern Ireland

**Keywords:** Computer vision, Deep learning, Small sample, Gestational age

## Abstract

A baby's gestational age determines whether or not they are premature, which helps clinicians decide on suitable post-natal treatment. The most accurate dating methods use Ultrasound Scan (USS) machines, but these are expensive, require trained personnel and cannot always be deployed to remote areas. In the absence of USS, the Ballard Score, a postnatal clinical examination, can be used. However, this method is highly subjective and results vary widely depending on the experience of the examiner. Our main contribution is a novel system for automatic postnatal gestational age estimation using small sets of images of a newborn's face, foot and ear. Our two-stage architecture makes the most out of Convolutional Neural Networks trained on small sets of images to predict broad classes of gestational age, and then fuses the outputs of these discrete classes with a baby's weight to make fine-grained predictions of gestational age using Support Vector Regression. On a purpose-collected dataset of 130 babies, experiments show that our approach surpasses current automatic state-of-the-art postnatal methods and attains an expected error of 6 days. It is three times more accurate than the Ballard method. Making use of images improves predictions by 33% compared to using weight only. This indicates that even with a very small set of data, our method is a viable candidate for postnatal gestational age estimation in areas were USS is not available.

## Highlights

• New small sample learning method combines photos/quantitative data, CNN & regression.
• Its application to GA estimation using newborns' photos (face/ear/foot) and weight
• In-depth analysis of data collected (& its effects) and the system (& its elements)
• Results improve current automatic & manual postnatal methods.
• Even with small dataset, viable postnatal estimation candidate in areas without USS

## 1 Introduction

According to the World Health Organisation (WHO), 10% of babies are born prematurely each year, amounting to over 15 million preterm babies annually [1]. Complications related to preterm birth remain the leading cause of death for children under 5 years [2], with over 1 million deaths just in 2013 [3]. Estimates suggest that over 75% of these deaths could be prevented with the right treatment [5].

Gestational age helps clinicians determine whether or not a newborn is premature and their degree of prematurity [4]. This estimation influences the treatment that the babies receive and could, consequently, result in suboptimal care and a poor outcome if the estimation is incorrect. In high-income countries, the gestational age of a baby is calculated prenatally with extreme accuracy thanks to early dating scans performed using USS and trained personnel [6]. However, in regions where USS cannot be deployed due to the remoteness of the area or lack of funding, the estimation of gestational age is a challenge. In these countries, in which the rate of premature births can reach up to 18% [2], the most widely used method is the Ballard Score, a manual scoring system that looks at neuromuscular and physical attributes of newborns. This method requires significant training and, even then, it is subjective and prone to errors, especially in low-income countries [7,8]. The Ballard Score is primarily based on visual analysis of a baby's features at different developmental stages. Thus, the opportunity for a computer-vision based analysis is promising. Nevertheless, the use of existing technology, such as pre-trained models, is not a suitable option due to a combination of three major reasons: 1) there are no pre-trained models for ear and foot classification, both of which are vital regions for the calculation of the gestational age, 2) the already pre-trained models for that use mostly use faces from children and adults, never newborns, and they are focused on age classification, not gestational age classification which is vastly different and much more nuanced.

We present a novel method for postnatal gestational age estimation that eliminated the subjectivity issues present in the Ballard Score. Our Small Sample Deep Learning approach was particularly suited for small and skewed datasets, such as our 88-participant dataset. Our system combined Convolutional Neural Networks (CNNs) and linear regression. While the task is essentially a regression problem, our proposed approach reduced the output space of the Deep Learning component to five major categories (extremely preterm, very preterm, moderately preterm, term, and late term), each of which is predicted with a certain probability. These probabilities were then combined with the normalised weight of the babies using a simple linear regressor. Our method was particularly useful because it allowed us to maintain the fine-grained prediction required by the original regression task, while still being able to benefit from deep learning's ability to automatically learn features from the images. Results were quite promising, with an expected error of 6 days and a 30% improvement over prediction based on weight only.

**Fig. 1:**
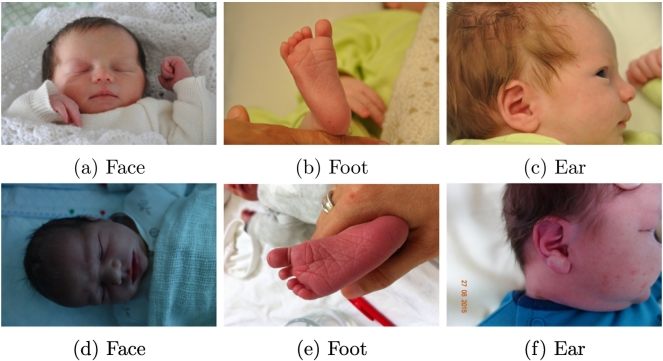
Example of newborn images from our database.

The contributions of this paper are:

1. A novel method for small sample learning which combines photographs and quantitative information in a two-stage process in which, first, broad classes are calculated via convolutional neural networks and, second, fine-grained classes are then predicted using the output from the convolutional neural networks.
2. An application of such method for the problem of gestational age estimation, in which the photographs used are from a newborn's face, ear and foot and the quantitative information is the weight. We also present an in-depth study of the system when different regressors and different combinations of the data are used. We have experimented with Linear Regression, Random Forest Regression, linear SVR and polynomial SVR.
3. A larger, more developed dataset for gestational age estimation. The new version of the dataset, The GesATional Dataset V2 (see Fig. 1), contains 130 participants versus the 88 participants from [9]. It still remains skewed, with over 50% of the dataset contained in the categories of moderately preterm and term, but includes a larger number of images from the ear and face region, which were particularly challenging to record previously. Additionally, in-depth analysis and discussion of the characteristics of the data, the improvements of the method, and the experiments are presented.

Results show that even with data of only 130 babies, we are able to segment and localise the regions of interest (face, foot and ear) with a Ballard Score of 0.91, 0.88 and 0.90, respectively. This entails a respective improvement of 18%, 8%, and 23% over the results presented in [9]. In terms of gestational age estimation, we are able to improve the current manual state-of-the-art, the Ballard Score, by 21.8%, resulting in gestational age estimations accurate to 7.98 days RMSE, and with 6 days of expected error. Additionally, we also improve the current automatic state-of-the-art methods by almost one day.

This paper is structured as follows: Section 2 gives an overview of the relevant literature in the fields of gestational age estimation, pre and postnatal, general age estimation, and segmentation. Section 3 describes in-depth the dataset (GestATional Dataset v2), which has data from 130 babies. We also discuss the major challenges that we have faced during the data collection and analysis process. Section 4 describes in detail the characteristics of the improved two-step method developed, while Section 5 summarises the experiments that were undertaken, and Section 6 discusses the results obtained and compares them with our previous results. Finally, Section 7 summarises this paper and discusses future work.

## 2 Related work

In this section, we briefly review relevant literature on three main topics: Gestational Age Estimation, Age Estimation and Image-based Segmentation.

### 2.1 Gestational Age Estimation

There are three major methods currently in use for gestational age estimation: Ultrasound Scans (USS), Last Menstrual Period (LMP), and clinical assessments such as the Ballard Score [10]. USS are prenatal and accurate to within a day if performed early in pregnancy (i.e. during the first trimester, [6]). However, USS machines are less accurate if used outside the first trimester, they are expensive, and cannot be deployed to many rural areas [10]. Additionally, they require trained personnel to use them, and report biased estimations for very large or small foetuses [10].

**Fig. 2:**
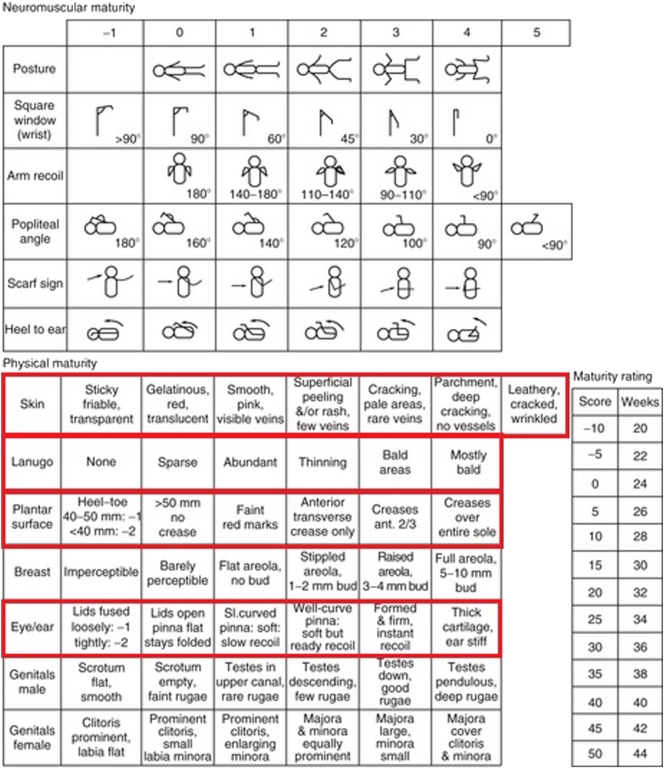
The Ballard test [7]. Neuromuscular and Physical information is measured to give an estimate of the gestational age of the newborn postnatally. Physical information marked in red (skin, lanugo, plantar surface and eye/ear) can be easily extracted and measure using computer vision.

The LMP and Ballard methods, in comparison, are low-cost and easy to deploy [11,12]. The LMP is an antenatal method that calculates the gestational age of a baby from the mother's last menstruation until the birth of the baby [10]. On the other hand, the Ballard Score (shown in Fig. 2) is a postnatal method that looks at two different sets of measurements regarding the newborn: Neuromuscular and Physical criteria. Neuromuscular criteria include posture, square window, arm recoil, popliteal angle, scarf sign, and heel to ear measurements, while Physical criteria include skin, ear/eye, lanugo hair, plantar surface, breast bud and genital developmental assessments. However, these two methods can be very inaccurate. Using the LMP entails estimation problems due to uncertainty, very often due to bleeding not related to periods or delayed ovulation. The LMP method can also be influenced by irregular menstruation due to nutritional issues or maternal disease, often common in low and medium income countries [10]. The Ballard Score is reported to be subjective, dependent on the clinicians' experience and, overall, inaccurate [7]. A clear example of this can be found in [8], where trained clinicians administered the Ballard exam to over 1000 (mostly term) newborns and obtained errors between 4 and 5 weeks when compared with USS scans.

Few researchers have attempted to develop methods of automatic gestational age estimation. Most research is in Anthropology and focuses on using simple techniques, like linear regression, and measurements of skeletal remains or brain weight [13], which are nearly impossible to obtain in rural settings.

This paper presents an alternative to these methods. It is automatic and combines the objectivity and accuracy of the USS scans, and the accessibility of the LMP and the Ballard Score. In an effort to automate and objectify the manual gestational age estimation process, the extraction of core Ballard's Physical criteria will serve as the motivation of our system, as most of them (shown in red in Fig. 2) can be easily measured in a much more objective manner using computer vision.

### 2.2 Automatic Age Estimation

Automatic Gestational Age Estimation is in many ways related to Automatic Age Estimation. Here we provide a general overview of popular methods for age classification. The problem of Age Estimation has recently gained popularity within the Computer Vision community, with many databases released and challenges organised [14,15].

Being essentially a Computer Vision task, the current state-of-the-art methods use Deep Learning in one form or another [16,17,[19], [20], [21]]. However, one thing that separates our problem from traditional age estimation, and consequently makes these methods unsuitable, is the ease with which data can be obtained. In [15], participants were given thousands of images from different people, while [21] needed tens of thousands of images to apply Convolutional Neural Networks (CNNs) to classify images according to age. Similarly, [16] and [17] report their findings in FG-NET [18] and MORPH [14], which collect images from over one thousand and five thousand individuals, respectively. In stark contrast, our current dataset consists of only 130 babies. For this reason, we decided to apply the techniques of Deep Learning, but had to overcome the challenge to create a new method that would work for the type of real data that we were collecting (small and skewed samples).

### 2.3 Image-based Segmentation

A vast amount of research has been done in the area of segmentation, particularly in the area of biomedical imagery [22,23]. Again, and unsurprisingly, the current state-of-the-art uses Deep Learning. One of the most popular contemporary approaches is the Fully Convolutional Neural Network (FCN, [24]). FCN approaches segmentation as a per-pixel classification problem and modifies traditional CNNs by substituting the final fully-connected layers for 1×1 convolutions. Due to their robust and accurate results in problems such as object recognition [24], we have decided to apply FCNs in the first stage of our system.

## 3 The GestATional Dataset

This paper presents an updated version of The GesATional Dataset (referred to as Version 2 or V2 of the dataset), which originally had 88 participants [9,31]. Version 2 of the dataset includes participants recruited from October 2015 to October 2017. It has been expanded for this paper to include information from 130 participants. This entails a 42% increment in terms of participants recruited. Recruitment and data collection were crucial for this project. While the ultimate goal is to deploy our image-based gestational age estimation system in areas without USS, in order to obtain ground truth data to learn our algorithms we needed to recruit participants for whom the gestational age was determined by USS, our gold standard. Participants were sorted into five different classes according to their degree of prematurity using a standard World Health Organisation categorisation scheme. These classes are shown in Table 1.

**Table 1:** Comparison in data distribution (participants and photographs) between versions 1 and 2 of the GestATional dataset according to five classes of gestation, from extremely premature to late term. w stands for gestational age in weeks.

	GestATional v2				GestATional v1 [9]			
	Babies	Images			Babies	Images		
		Face	Foot	Ear		Face	Foot	Ear
Extremely (≤ 28)w	13	26	103	13	8	26	46	13
Very (28 to 32 w)	25	91	188	64	22	73	161	49
Moderate (33 to 36 w)	38	280	295	207	22	86	119	53
Term (37 to 40 w)	33	214	276	172	18	68	140	50
Late (≥ 40w)	21	95	214	83	18	50	166	17
**Total**	**130**	**706**	**1074**	**539**	88	303	632	207

**Fig. 3:**
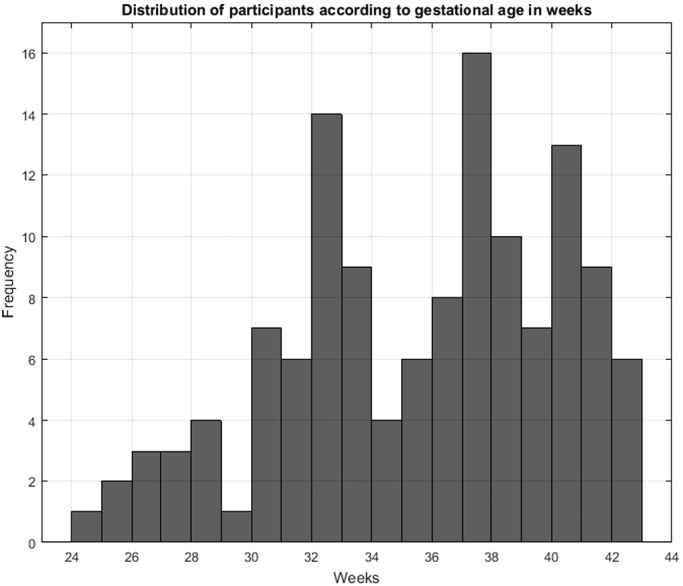
Distribution of gestational ages from the 130 participants of The GestATional Dataset v2.
**2. Case Report Forms (CRF):** with relevant information such as the gestational age of the baby, days of life at the time of the visit, current weight, Ballard Score as performed by the clinical research team in charge of recruiting participants (blinded to the gestational age of the baby), the medical history of the mother, and information about the delivery. The information on this document was used to collect the ground truth for the age estimation. All data was anonymised to guarantee that information could not be used to trace participants.

Participants were recruited by clinical staff at our local hospitals (Nottingham University NHS Trust, Nottingham, UK). Clinical researchers approached parents of newborn babies on the maternity ward and the neonatal intensive care unit. Ethics approval for this study was obtained from the National Health Service in the UK (NHS ethics committee approval, ref. 15/EM/0173), and from the School of Computer Science at the University of Nottingham. After informed consent had been taken, data collected from participants resulted in two sets of data:

**1. Images:** of the participant's face, foot and ear. In some cases, particularly in the case of newborns aged 28 weeks of gestation or less (extremely preterm), who are connected to machines, some of these images were difficult or impossible to obtain. Of the 130 participants recruited to date, 126 supplied foot images, 116 supplied ear images and 115 supplied face images. Additionally, each participant had between 2 and 10 images taken from each different body part, depending on the risk involved in taking them. More information about the number of babies and the number of photographs from each class can be found in Table 1. Furthermore, statistics from the participants can be found in Table 2 and a distribution of the participants according to their gestational age in weeks can be found in Fig. 3.

**Table 2:** Mean, Median, Minimum, Maximum and Standard Deviation of the gestational ages of the participants in weeks. Class-wise and overall statistics are shown.

	Mean	Median	Min	Max	Std.
Extremely	26.55	26.57	24.43	28	1.15
Very	30.86	31	29.28	32	0.87
Moderately	34.78	35	32.14	36.86	1.62
Term	38.39	38.28	37	40	1.00
Late	41.06	41.14	40.14	42	0.62
**All data**	**35.17**	**36.14**	**24.43**	**42**	**4.54**

### 3.1 Data annotation

Since part of our system first needs to automatically locate the different body parts within the image, landmarks were annotated in the images, which were then used to train and test our segmentation step. To annotate version 2 of the dataset, we employed 5 annotators who spent over 500 h of work in the span of six months.

**Fig. 4:**
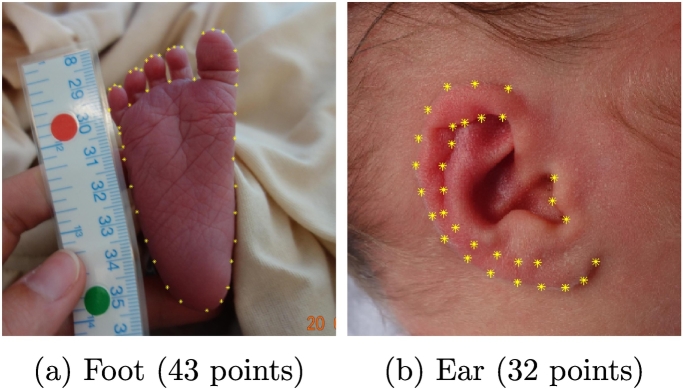
Examples of an annotated ear and foot photographs.

Foot images required 43 points, while face images needed 68 points and ear images needed 32 points. An example of an annotated foot and an annotated ear are shown in Fig. 4.

### 3.2 Challenges

**Fig. 5:**
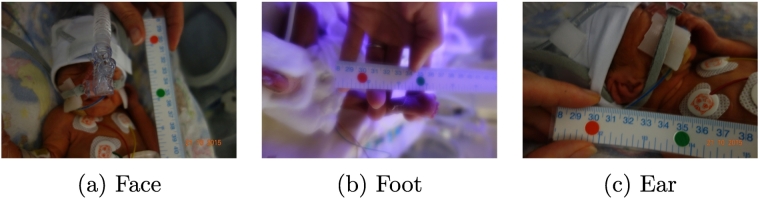
Examples of challenging images. Major challenges include blurry images due to incubator conditions or heavy occlusion due to babies receiving treatment.

The sensitive nature of this project and the characteristics of the participants needed resulted in a number of challenges that affected both the data collection stage and computation stage. The team behind the project encountered two main challenges:

**1. Recruiting babies:** Recruiting moderately preterm, term, and late babies was straightforward and successful, since the babies were not undergoing any invasive treatment and taking photos did not incur any additional stress for them. However, understandably, parents of extremely and very preterm babies were often too worried about their child and about potentially interrupting their serious treatment to take photographs. As a result, despite our best effort our database is somewhat skewed towards moderately preterm, term and late babies.
**2. Taking high-quality photos:** Not only were extremely and very preterm babies difficult to recruit, they were also hard to photograph, due to the babies being inside incubators and connected to machines. This resulted in members of our team not being able to collect images from babies belonging to these categories or in the images being blurry or heavily occluded by clothes, patches, or machines. A visual example of the effects of this challenge is shown in Fig. 5. These photographs were too blurry or had too much occlusion to be suitable to be used in our dataset. The effects of occlusion were particularly noticeable when photographing faces (due to babies being connected to machines) and ears (due to babies wearing hats to maintain heat). Fig. 5a and c exemplifies the type of occlusion that many photographs of extremely and very premature babies had.

The effects of these challenges are shown in Table 1, where the differences between extremely preterm babies (13 participants, adding up to a total of 13 ear images) and moderately preterm babies (with 25 participants and 64 ear images) are shown in terms of the number of images collected.

## 4 Small Sample Learning

Our Small Sample Deep Learning method presented in this paper can be divided into two stages:

1. Segmentation: which uses FCNs [24] to localise the regions of interest (foot, ear and face) within an image.
2. Gestational Age Estimation: Which uses a bounding box around said regions of interest with a combination of CNNs and Regression to generate a prediction on the gestational age of a baby.

### 4.1 Segmentation

**Fig. 6:**
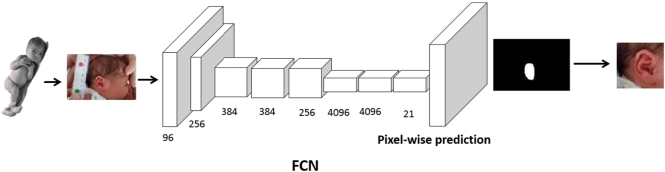
In the first step of our framework, FCNs [24] are used to segment the images and find regions of interest. Newborn's stock photo from [26].

The first stage of the system, shown in Fig. 6, is carried out using Fully Convolutional Neural Networks (FCNs) [24]. We have used FCNs to segment the images taken by our team and localise where the foot, ear and face are within each image. FCNs are currently competitive with state-of-the-art methods for segmentation [24]. They use the same architecture as a VGG network [25] with one major difference: the traditional fully-connected layers are replaced by 1×1 convolutions. This allows them to provide a per-pixel classification and, consequently, segment the original image.

As shown in Fig. 6, the input of this stage are the pre-processed images from our dataset. The output of the FCNs are binary masks in which pixels that were predicted as part of the body parts that were being classified are activated. The ground-truth used in this stage are binary masks created using the polygons that resulted from manual annotations. In these masks, pixels equal to 1 belong to feet, faces or ears and pixels with a value of 0 are part of the background.

Once the predicted segmentation masks were obtained, a simple post-processing stage was carried out to isolate the activated regions that belonged to either the ear, face and foot of the babies. Since some of the images showed the clinician's hands or other parts of the baby, such as their legs, we found that some patches of flesh from these regions were sometimes predicted as ear, face and foot. In a post-processing step the region with the largest area of activated pixels was retained while discarding any other spurious regions of activation. This successfully removed many incorrectly predicted pixels.

### 4.2 Gestational Age Estimation

The second stage of our framework is the Gestational Age Estimation stage. For this stage, we created a new architecture of CNNs, called CVL17 [9], specifically designed for small and imbalanced datasets. In [9], we described novel method of combining this architecture with linear regression to obtain an estimation of the ages in days. The advantages of this process were threefold. It allowed us to:

• Take advantage of current state-of-the-art methods (CNNs) to learn features, even when our input data does not fit the criteria that CNNs usually require (i.e. large amounts of data and balanced classes).
• Combine visual information and anthropometric measures, such as the weight of the babies in the decision-making process.
• Provide an estimate of the gestational age in weeks, instead of classes. These results are, by definition, more fine-grained.

In this paper, we present an improvement on our original method based on experiments with more sophisticated regressors. In particular, we have experimented with Random Regression Forests, Linear Support Vector Regression (SVR) and Polynomial Support Vector Regression, out of which SVR obtained the most successful results.

**Fig. 7:**
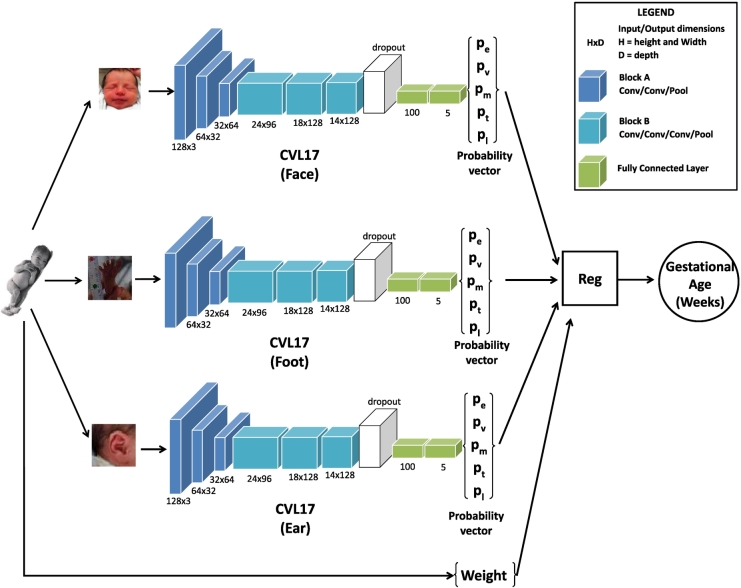
Overview of the age estimation process when feet, face and ear photographs are combined. *p*_*e*_ is the probability of the test image belonging to the extremely preterm class, *p*_*v*_ is the probability of the test image belonging to the very preterm class, *p*_*m*_ is the probability of the test image belonging to the moderately preterm class, *p*_*t*_ is the probability of the test image belonging to the extremely term class, and *p*_*l*_ is the probability of the test image belonging to the late term class.
  First, there are three blocks of type A, followed by three blocks of type B. All convolutions involved in the architecture are 3×3 convolutions. All blocks reduce the dimension of the input feature vector by half. Then, after a dropout layer, there are two fully-connected layers. The first one has 100 outputs and the second one has 5, which map to the 5 classes we want to recognise. We use a SoftMaxLoss layer to measure error. Since we are using images from three different regions, we train separate CNNs for the feet, face and ear.
**II. Regression:** Once the CNNs have been trained, the test images are propagated through the network and the probabilities obtained as the output of the CNN are stored for each of the babies in the test set and each of the regions. This produces a 5×1 probability vector, as shown in Fig. 7, where *p*_*e*_ is the probability of the test image belonging to the extremely preterm class, *p*_*v*_ is the probability of the test image belonging to the very preterm class, *p*_*m*_ is the probability of the test image belonging to the moderately preterm class, *p*_*t*_ is the probability of the test image belonging to the extremely term class, and *p*_*l*_ is the probability of the test image belonging to the late term class. This process was repeated using subject-independent 5-fold cross-validation to obtain predictions for the whole dataset in a manner that avoids overfitting. The 5-dimensional image-based probability vectors are combined with the normalised weight of the participants and used as the input of a regressor, which outputs an estimate of the gestational age of the babies in weeks.
  After obtaining very promising results with a simple linear regressor in [9], we decided to explore more complex regressors, which have consistently obtained better results in popular computer vision problems. In this paper, we present extensive experiments and an in-depth analysis on the performance of our method when using Random Regression Forests [30], Linear Support Vector Regression and Polynomial Support Vector Regression [29].

Training of our Small Sample Deep Learning structure consists of two phases:

**I. Convolutional Neural Networks:** CNNs were used to classify images into five coarse classes (presented in Section 3). Since our dataset was relatively small and there was a high imbalance between babies born before 28 weeks and babies that were 35 weeks or older, we decided against grouping participants according to their gestational age in weeks. This would have resulted in 14 classes (from 26 weeks-old to 40 weeks-old) with extremely small sample sizes. In fact, some classes would even have had no examples at all. Grouping participants into five classes guaranteed more populated classes with a more balanced distribution of images.
  For the purpose of training small sets of data, we created our own network using Caffe: CVL17, designed to work with a limited and skewed set of data: it is “deep” enough that features can be learned and, at the same time, it is “shallow” enough that it can classify images confidently. As shown in Fig. 7, CVL17 takes 128×128×3 RGB images as input and it is formed of two types of building blocks:
  • Block A: convolution, relu, convolution, relu, pooling.
  • Block B: convolution, relu, convolution, relu, convolution, relu, pooling.

## 5 Experiments

This paper presents in-depth experiments, analysis, and discussion on the performance of our system and dataset. Since two main variables changed (the size of our dataset, now with over 40% more data, and the choice of regressor, now four different possibilities), we decided to test the effect of each one separately. As a consequence, we carried out all of our testing scenarios in both versions of the dataset.

For all of our testing scenarios, we used 5-fold subject-independent cross-validation. This guaranteed no overlap between photos from babies used during training and photos from babies used during testing. We used two-stage cross-validation. In the first stage, we do subject-independent cross-validation to generate predictions made by the CNN. We then treat these predictions as the features of our second stage, where we evaluate the linear regressors again using subject-independent cross-validation.

The purpose of these experiments was to prove the following hypotheses:

1. Our combined methodology outperforms the use of each of its components (end-to-end CNNs and regression) separately.
2. FCNs can be used to accurately locate faces, feet and ears within the images of our database.
3. Increasing the number of images directly results in an overall improvement across all metrics studied.
4. Combining the normalised weight of a baby and visual information can improve the current state-of-the-art in terms of postnatal methods.
5. The use of more sophisticated regressors can improve the current automatic results (compared to those reported in [9]).

Hypothesis 1 was tested with an ablation study in which we compared each element of the methodology separately. Hypotheses 2 to 5 were tested by experimenting with four possible regressors (Linear Regressor, Random Regression Forests, Linear Support Vector Regressors, and Polynomial Support Vector Regressors) in both versions of the dataset (V1, with 88 participants, and V2, with 130). All results from these experiments are shown in Section 6.

Due to the small number of images in our dataset, we needed the characteristics of our images in terms of size, orientation and perspective to be as similar as possible. Assuring that all images had the same properties would diminish and even eliminate any negative effect that variations on size and layout could bring into the segmentation and estimation process. However, circumstances not always allowed photographs to be taken under the exact same conditions. Consequently, the raw photographs taken by our team were pre-processed according to:

1. Size: With a size of 4 MB, raw images were too large to be used as the input of FCNs. To solve this, images were resized to 10 KB.
2. Orientation: Images had inconsistent orientations. To solve this, we rotated all images until they were landscape images with the captured body part in an upright position.
3. Perspective: We originally intended to capture the right foot and right ear from all babies. However, due to some babies undergoing treatment, this was not always possible and photographs of their left foot or ear were taken. To solve this, images with left ears or feet were horizontally flipped.

After this pre-processing step, all images had the same characteristics and they were ready to be segmented.

Using the masks obtained from the segmentation step, bounding boxes were created around the largest region of activated pixels within the masks. Bounding boxes were centred around these blobs and resized to 128×128 pixels.

**Fig. 8:**
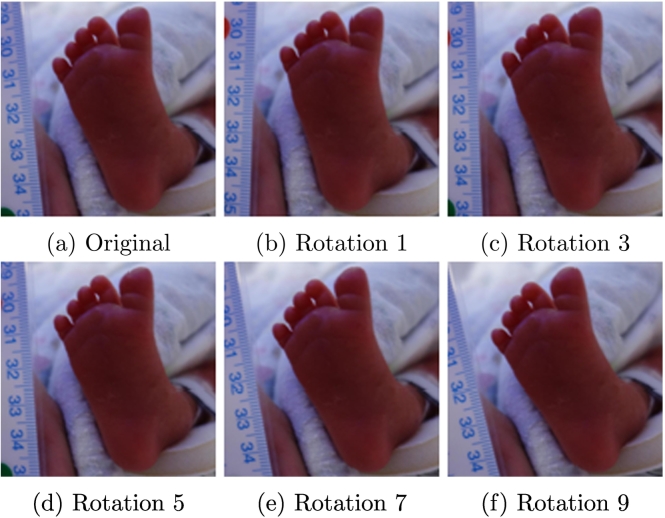
Examples of 6 rotations from the foot region of an extremely premature baby.

Additionally, we carried out some data augmentation to balance the dataset. As shown in Section 3, both versions of our dataset are limited and quite skewed. While deep learning methods represent the state-of-the-art in terms of classification methods, they require large quantities of data to perform adequately. Therefore, we decided to rotate the bounding boxes within the images between −10 and 10 °. Depending on the gestational age of the participant, a different number of rotations were added to our training dataset in an effort to balance instances from all classes. Extremely preterm babies had 10 rotations added to the dataset, very preterm babies had four rotations added, and moderately, term, and late preterm babies had two rotations added. This way, the final number of images that were used for training were more balanced. Examples of six rotations for the foot of an extremely preterm baby are shown in Fig. 8.

### 5.1 Metrics

Segmentation is measured using the Jaccard Index, while Age Estimation uses RMSE.

#### 5.1.1 Jaccard Index

The Jaccard Index is widely used in segmentation problems, particularly in Biology [27]. It measures the intersection over the union of two sets of points [28]. It is calculated with:

$$J(P,GT)=\frac{\left|P\cap GT\right|}{\left|P\cup GT\right|}$$

Where *P* is the prediction (pixel set returned by FCNs as belonging to body parts), and *GT* is the ground-truth.

#### 5.1.2 RMSE

The Root Mean Square Error was used because it allows us to measure the error of our predictions in the same units as the ground-truth, weeks.

$$RMSE=\sqrt{\frac{1}{n}\sum_{i=1}^{n}{\left(y_{i}-{ŷ}_{i}\right)}^{2}}$$

Where *y*_*i*_ is the gestational age of the *ith* baby and ${ŷ}_{i}$ is the prediction for the gestational age of the *ith* baby according to our linear regressor.

### 5.2 Setup

Experiments were carried out on a machine using an NVIDIA's Titan X GPU. For the Segmentation step, we ran each stage of the FCNs for 30,000 iterations (93 min) with a learning rate of 10^−4^ and a step of 0.9. For the Gestational Age Estimation step, we trained each CNN for 20,000 iterations. Training with V1 of the dataset took 5 h and 12 min, while training with V2 of the dataset took 5 h and 50 min.

## 6 Results and discussion

Results from our segmentation experiments are shown in Table 3, which contains the mean and median Jaccard Index obtained when segmenting each region with and without post-processing. Furthermore, results from the gestational age estimation stage are shown in Table 4, Table 5, Table 6.

**Table 3:** Mean and Median Jaccard Index for segmentation of Face, Foot and Ear on The GesATional Dataset V1 and V2.

	GestATional V1				GestATional V2			
	No Post Proc		Post Proc.		No Post Proc		Post Proc	
	Mean	Median	Mean	Median	Mean	Median	Mean	Median
Face	0.73	0.78	0.73	0.78	0.91	0.93	0.91	0.93
Foot	0.79	0.85	0.79	0.86	0.88	0.91	0.88	0.91
Ear	0.67	0.77	0.69	0.78	0.90	0.91	0.90	0.91

Table 4 shows an ablation study in which we test each element of our methodology separately and compare it to our novel methodology for each region separately. These elements are: 1) CVL17 as an end-to-end classifier with 19-classes (from gestational ages of 24 weeks to 42 weeks), and 2) CVL17 as an end-to-end regressor.

**Table 4:** Ablation study for V1 (88 participants). End-to-end 19-class CNNs and Regression CNNs perform poorly on their own. Our method, which combines both strategies vastly improves the results even when only considering regions (face, foot, ear) separately.

	End-to-End CNN		Regression CNN		Small Sample Learning	
Data	RMSE	Std E	RMSE	Std E	RMSE	Std E
Face	32.31	0.42	24.5	5.34	3.91	2.23
Foot	18.74	0.63	22.79	2.98	2.66	2.22
Ear	20.64	2.51	21.67	3.21	3.35	1.97

Finally, Tables 5 and 6 have shown the comparative results of our method in V1 and V2 of the dataset, respectively. Note that in all of these tables, Ft is Foot, F is Face, E is Ear and W is the normalised Weight. We reported results using: Linear Regression, Regression Random Forests (RRF) with 950 trees, Linear SVRs and third-degree polynomial SVR. Through cross-validation, we tested RRF from sizes 1 to 1500 and found 950 trees to be the optimal configuration. Furthermore, we found third-degree polynomials to yield the optimal results after testing SVR kernels from second to eight degrees.

**Table 5:** Gest. Age Estimation on The GestATional Dataset V1 (88 participants). Baseline results (Weight and Ballard) are shown in bold and italics. Our best result (in bold) improves both. Ft is Foot, F is Face, E is Ear and W is the normalised Weight.

	Lin-Regression		RRF		Linear SVR		Pol-SVR	
Data used	RMSE	Std E	RMSE	Std E	RMSE	Std E	RMSE	Std E
***Weight***	***1.50***	***1.00***	***1.25***	***1.03***	***1.92***	***1.15***	***1.77***	***1.05***
***Ballard***	***3.57***	***2.27***	***3.57***	***2.27***	***3.57***	***2.27***	***3.57***	***2.27***
Ballard S	3.72	2.27	1.92	1.22	2.62	1.99	5.07	3.22
Posture	4.14	2.09	3.25	2.03	3.56	2.09	3.48	2.25
Face	3.91	2.23	3.11	2.21	3.20	2.13	2.95	2.20
Foot	2.66	2.22	3.38	2.30	3.42	2.06	3.48	2.25
Ear	3.35	1.97	3.75	2.00	3.84	2.32	3.90	2.79
Face + Weight	1.63	1.32	1.82	1.38	1.75	1.41	1.26	1.83
Foot + Weight	1.40	1.25	1.93	1.45	1.26	1.04	1.33	1.08
Ear + Weight	1.46	1.33	2.03	1.29	1.32	0.94	1.44	1.06
Face + Foot	2.81	2.67	2.98	2.96	2.62	2.03	3.09	2.74
Face + Ear	3.24	2.83	3.10	2.12	3.14	2.30	3.44	2.32
Foot + Ear	3.67	2.78	3.31	2.24	3.38	2.17	3.42	2.68
F + Ft + E	3.17	2.88	3.02	2.00	2.68	2.13	2.91	2.75
F + Ft + W	1.32	1.01	1.78	1.34	1.20	0.91	1.83	1.35
F + E + W	1.15	0.89	1.84	1.21	1.21	0.82	1.56	1.55
Ft + E + W	1.23	1.06	1.93	1.35	1.29	0.97	2.42	2.02
**F+Ft+E+W**	1.29	0.99	1.84	1.35	**1.12**	**0.87**	2.36	3.14

**Table 6:** Gest. Age Estimation on The GestATional Dataset V2 (130 participants). Baselines shown in bold and italics. Our best result (in bold) improves both. Ft is Foot, F is Face, E is Ear and W is the normalised Weight.

	Lin Reg		RRF		Lin-SVR		Pol-SVR	
Data used	RMSE	Std E	RMSE	Std E	RMSE	Std E	RMSE	Std E
***Weight***	***2.4***	***1.67***	***1.47***	***1.62***	***1.39***	***1.01***	***1.25***	***0.83***
***Ballard***	***4.55***	***2.42***	***4.55***	***2.42***	***4.55***	***2.42***	***4.55***	***2.42***
Ballard S.	4.42	3.48	1.93	1.25	5.02	3.24	4.80	3.77
Posture	4.13	2.30	3.22	1.89	3.48	2.25	4.8	3.7
Face	3.31	2.78	3.32	2.47	3.21	2.40	3.32	2.69
Foot	3.48	2.57	3.48	2.66	3.31	2.65	3.34	2.79
Ear	2.70	2.22	2.75	2.17	2.7-	2.02	2.73	2.08
Face + Weight	1.5	1.35	1.91	1.48	1.43	1.31	1.40	1.71
Foot + Weight	1.82	1.43	2.10	1.63	1.73	1.40	1.73	1.28
Ear + Weight	1.41	1.03	1.77	1.30	1.39	0.97	1.31	1.06
Face + Foot	3.39	3.05	2.88	2.34	3.02	2.45	2.97	3.53
Face + Ear	3.19	3.04	2.83	2.10	2.72	2.16	2.89	2.57
Foot + Ear	2.68	2.2	2.05	2.82	2.62	2.15	3.00	3.35
F + Ft + E	3.09	2.85	3.12	1.99	3.06	2.17	3.84	2.92
F + Ft + W	1.34	1.00	1.86	1.57	1.47	1.37	1.67	1.31
F + E + W	1.17	0.9	1.69	1.28	1.16	1.1	1.82	2.15
Ft + E + W	1.3	1.01	1.9	1.28	1.31	1.11	2.00	1.69
**F+Ft+E+W**	1.22	1	1.65	1.23	**1.14**	**0.88**	1.8	1.45

Results from the segmentation step showed that FCNs improved their performance when segmenting Version 2 of the dataset. By increasing the size of the dataset, we achieved a Jaccard Index of 0.88 for feet (11% more accurate) and 0.91 for face segmentation (24% more accurate). However, the most significant improvement occurred in the segmentation of ears, for which FCNs obtained a Jaccard Index of 0.9 for ears, entailing over a 34% increase in segmenting accuracy.

Another point of interest was discovered when we compared the performance of FCNs in both versions of the dataset. While Version 1 benefited from having a post-processing step that removed smaller blobs (considered noise) and only retained the largest blob within a masks, once the dataset increased in size, this step had become unnecessary, as the FCNs did not predict these smaller blobs any more. In summary, the increase in the size of the dataset allowed for faster segmentation and an improvement of 18%, 9% and 23% in terms of the Ballard Score when segmenting face, feet and ear, respectively.

**Fig. 9:**
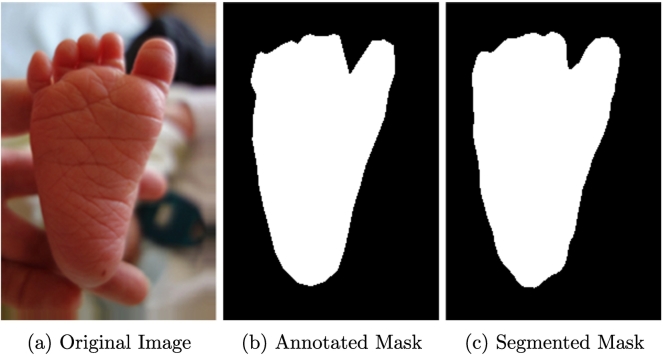
Foot segmentation. FCN result in c) in much smoother and a better fit than manually-annotated mask in b), outperforming ground-truth).

Additionally, the predicted masks were more accurate than the simple masks that we used, as shown in Fig. 9. These results were sufficiently accurate to localise the body parts of interest, and to obtain their largest dimension (height or width), which is the most important information to generate bounding boxes for CNN training. The similarity between the median and mean of the Jaccard Index indicates that the results are consistent across all images in the different image datasets.

Results from the ablation study proved our original hypothesis that established that our novel method which uses CNNs for broad classes and then regression to fine-tune predictions outperforms the use of each of those elements separately. This is a direct consequence of the small number of samples in our dataset.

Looking closely at the results obtained at the gestational age estimation stage and comparing the performance of both datasets and all four different regressors, interesting points were raised.

**Fig. 10:**
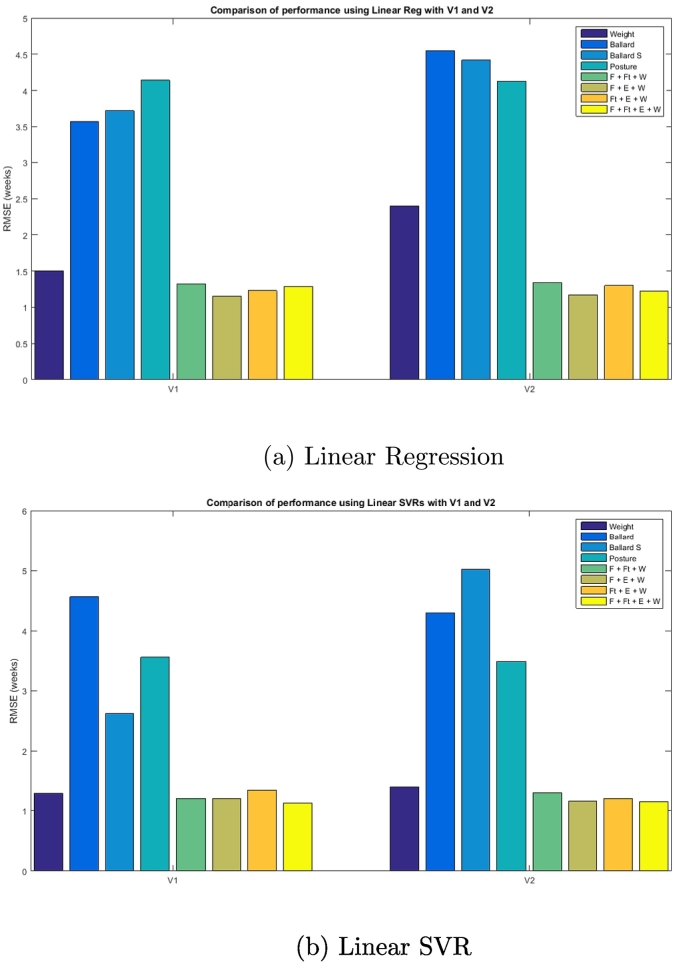
Manual vs automatic methods with a) Lin. Reg. (state-of-the-art) and b) Lin. SVR (new architecture presented in this paper) with both versions of the dataset.

First of all, we were able to improve current manual postnatal methods, such as the Ballard score and weight regression. A comparison of the performance of these methods for both datasets when using linear regression and linear support vector regression can be found in Fig. 10b. As shown in these figures, both types of regressors combined with photographs and normalised weight obtain dramatic improvements over manual methods. Interestingly, this improvement, which surpasses 30% in some cases, happens regardless of the combination of regions used and in both versions of the dataset.

Secondly, and more importantly, we also surpassed the current state-of-the-art method for automatic postnatal gestational age estimation, presented in [9]. We improve [9] by 0.03 in the case of Version 1 of the dataset and 0.01 in the case of Version 2 of the dataset. Using V1, we were able to achieve a RMSE of 1.12 with a standard error of 0.87 or 6.09 days. Using V2 of the dataset, we obtained an RMSE of 1.14 with a standard error of 0.88 (6.16 days). Further analysis of the results, also brought to our attention that both of these new state-of-the-art results where obtained using support vector regression with a linear kernel. In general, Linear SVR outperformed all other methods in all categories. On the other hand, regression forests and third-degree polynomial support vector regression generally obtained less accurate results.

**Fig. 11:**
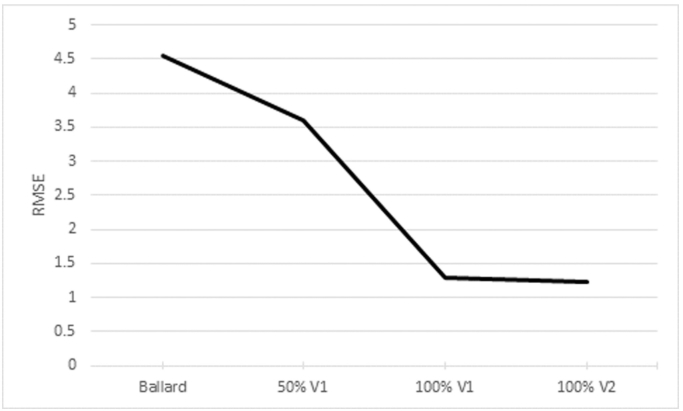
Average RMSE across 5 folds when using 0% of the images (Ballard Score), 50% of V1, 100% of V1 and 100% of V2. The input in this example is the combination of all regions and the weight of the newborns.

Another interesting point is that, in general, the increase in training data improved or maintained the performance of our automatic method but it made manual methods worse. The characteristics of the training samples, especially the size and quality of the samples, are crucial for accurate classification. Since there are many possible variations within our participants, we hypothesized that an increase in the dataset would entail an increase in accuracy, in which experiments show to be true. An example of this is shown in Fig. 11, where we report the RMSE of the estimations made using 0% of the training set (i.e. using the Ballard Score), and then using 50% of V1, 100% of V1 and then 100% of V2 with all three regions and the weight as input. As can be seen, as the size of the training set increases, so does the accuracy, while the standard deviation decreases. This is further supported by comparing overall performances between V1 and V2, as shown in Fig. 10b.

Futhermore, it can be seen by comparing the results for V1 and V2 in Tables 5 and 6 that V2 of the dataset is harder to predict than V1, as exemplified by the higher RMSE errors and standard error deviation when using the Ballard Score and the weight. This entails that V2, while larger, is harder to classify. Nevertheless, our automatic postnatal method is able to obtain results 41.7% and 21.9% more accurate than the weight and 65.3% and 74.9% more accurate than the Ballard Score for V1 and V2, respectively.

**Fig. 12:**
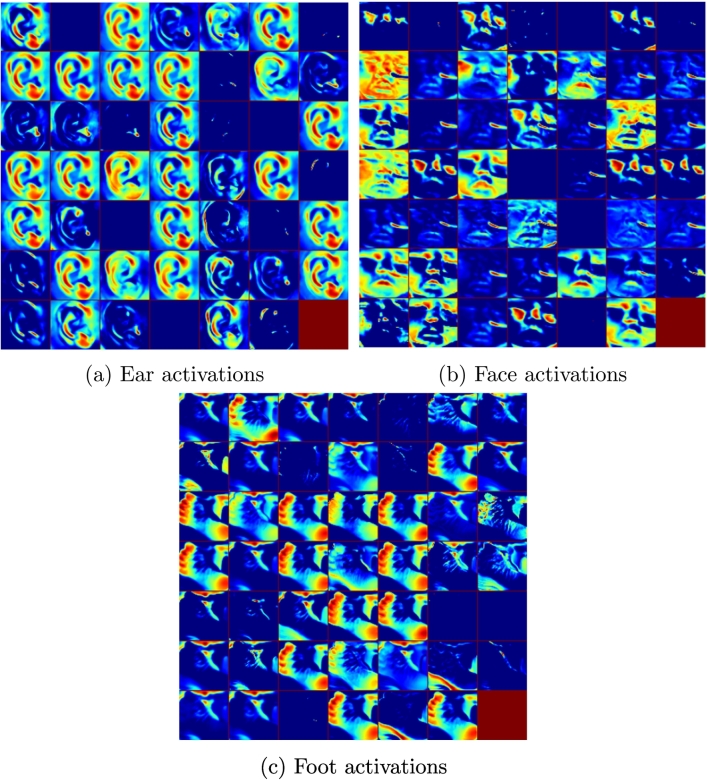
Ear, Face and Foot activations from the first convolution within our CVL17 network shown in a heat map.

Finally, by analysing the features learned at the network, it is clear that features at the lower levels of CVL17 closely match Ballard's physical measurements. This is exemplified in Fig. 12, which shows three random examples from the 48 activations obtained at the first convolution layer. It can be seen that regions related to the texture of the sole of the foot, the cartilage around the ears, the shape and openness of the eyes, and the texture of the skin are activated. These match the physical characteristics assessed in the Ballard Score (measurements marked in red in Fig. 2). In other words, the network is objectively extracting a subset of what clinicians are trained to assess when carrying out the Ballard Score, and it is using them in the classification process.

## 7 Conclusions and future work

The gestational age of a baby is crucial when determining the best treatment for a newborn, especially when born prematurely. We have extended work previously presented in [9] in which a system that estimates the gestational age of babies postnatally using photographs of their face, foot and ear was introduced. Our system has two steps: first, images are segmented using Fully Convolutional Neural Networks to find where the relevant body parts appear in the image. Second, CVL17, a Convolutional Neural Network, is used to classify the photographs according to five classes (extremely preterm, very preterm, moderately preterm, term, and late). The probability vectors that result from these CNNs are then combined with the weight of the newborn and used as the input of a regressor. This allows us to output an estimation of the gestational age in weeks, instead of classes.

In this paper, we have presented an improved version of this system which uses images from the feet, face and ear of 130 newborn babies and a combination of FCNs, CNNs and Support Vector Regressors, to calculate the gestational age of a baby with a RMSE of 1.14 and an expected error of 0.88 week. Results show that as the size of our dataset increases, automatic results vastly outperform manual measurements, such as the weight and the Ballard Score. This further positions our system as a potential alternative to postnatal manual methods commonly used in remote and underfunded locations where USS are not available and health care workers may not be trained in clinical assessment of newborns. Furthermore, when we analysed the features that are being learned at the lower levels of the network, it became clear that our network is, in essence, automatically learning the measurements that doctors are taught to measure in the Ballard Score such as skin texture, planar surface and ear cartilage formation. In other words, our system is able to efficiently and accurately learn what doctors are taught to assess without being affected by issues introduced by lack of experience or subjectivity.

Now that we have further evidence on the effect of the size of the dataset and the importance of the regressor used, future work will focus on exploring new and more sophisticated deep learning networks in order to improve our current results. We will also continue recruiting more participants with the aim of creating a database with equal amounts of images for the three regions (face, foot and ear) and all five classes of babies (extremely premature, very premature, moderately premature, term and late). For this, we are in the process of recruiting more participants during the next months, focusing on the more challenging categories (i.e. extremely and very premature babies). We are estimating that we will reach 150 babies by January of 2018, and plans are underway to recruit thousands of babies in India to test the method in a setting most likely to benefit from this approach.

This method could result in improved outcomes for the millions of vulnerable babies in low-middle income countries where clinical management is compromised due to incorrect or unknown gestational age at birth. Furthermore, by uploading this information to a cloud database we could obtain a more detailed picture of the populations where preterm birth is more prevalent.
